# Genome-wide association study of plasma levels of polychlorinated biphenyls disclose an association with the *CYP2B6* gene in a population-based sample

**DOI:** 10.1016/j.envres.2015.03.022

**Published:** 2015-07

**Authors:** Esther Ng, Samira Salihovic, P. Monica Lind, Anubha Mahajan, Anne-Christine Syvänen, Tomas Axelsson, Erik Ingelsson, Cecilia M. Lindgren, Bert van Bavel, Andrew P. Morris, Lars Lind

**Affiliations:** a Wellcome Trust Centre for Human Genetics, University of Oxford, Oxford, United Kingdom; b Department of Medical Sciences, Molecular Epidemiology and Science for Life Laboratory, Uppsala University, Uppsala, Sweden; c Department of Medical Sciences, Occupational and Environmental Medicine, Uppsala University, Uppsala, Sweden; d Department of Medical Sciences, Molecular Medicine and Science for Life Laboratory, Uppsala University, Uppsala, Sweden; e MTM Research Centre, Örebro University, 701 82 Örebro, Sweden; f Department of Biostatistics, University of Liverpool, Liverpool, United Kingdom; g Department of Medical Sciences, Cardiovascular Epidemiology, Uppsala University, Uppsala, Sweden

**Keywords:** Genome-wide association studies, Polychlorinated biphenyls, Cytochrome P450, Single nucleotide polymorphisms, Metabolism, Pollutants

## Abstract

**Background:**

Polychlorinated biphenyls (PCBs) are a group of man-made environmental pollutants which accumulate in humans with adverse health effects. To date, very little effort has been devoted to the study of the metabolism of PCBs on a genome-wide level.

**Objectives:**

Here, we conducted a genome-wide association study (GWAS) to identify genomic regions involved in the metabolism of PCBs.

**Methods:**

Plasma levels of 16 PCBs ascertained in a cohort of elderly individuals from Sweden (*n*=1016) were measured using gas chromatography–high resolution mass spectrophotometry (GC-HRMS). DNA samples were genotyped on the Infinium Omni Express bead microarray, and imputed up to reference panels from the 1000 Genomes Project. Association testing was performed in a linear regression framework under an additive model.

**Results:**

Plasma levels of PCB-99 demonstrated genome-wide significant association with single nucleotide polymorphisms (SNPs) mapping to chromosome 19q13.2. The SNP with the strongest association was rs8109848 (*p*=3.7×10^−13^), mapping to an intronic region of CYP2B6. Moreover, when all PCBs were conditioned on PCB-99, further signals were revealed for PCBs -74, -105 and -118, mapping to the same genomic region. The lead SNPs were rs8109848 (*p*=3.8×10^−12^) for PCB-118, rs4802104 (*p*=1.4×10^−9^) for PCB-74 and rs4803413 (*p*=2.5×10^−9^) for PCB-105, all of which map to CYP2B6.

**Conclusions:**

In our study, we found plasma levels of four lower-chlorinated PCBs to be significantly associated with the genetic region mapping to the CYP2B6 locus. These findings show that CYP2B6 is of importance for the metabolism of PCBs in humans, and may help to identify individuals who may be susceptible to PCB toxicity.

## Highlights

• This genome wide study identifies single nucleotide polymorphisms (SNPs) associated with levels of different PCBs.
• These SNPs are located in the Cytochrome 2B6 gene *(CYP2B6).*
• Patterns of association are complex.
• This study may help to identify individuals who may be susceptible to PCB toxicity.

## 1 Introduction

Polychlorinated biphenyls (PCBs) are a group of man-made lipophilic chemicals that have, over the years, been linked to a number of adverse effects (Brouwer et al., 1999). From the late 1920s until the late 1970s, PCBs were manufactured in a broad range of industrial applications (e.g. electrical equipment and insulation) where their exceptional chemical stability was found useful. Among the complete set of 209 different congeners of PCBs, twelve have been denoted as being “dioxin-like” because of their structural resemblance with 2,3,7,8-tetrachlorodibenzo-p-dioxin (2378-TCDD) (Van den Berg et al., 2006).

Although PCBs were banned decades ago, they are frequently detected in various biological and environmental samples around the world (Jones and de Voogt, 1999). In the general population, PCB contaminated food is considered to be the major source of exposure, and numerous studies have shown that foods with a high fat content such as fish, meat, and dairy products are of particular concern (Darnerud et al., 2006). Human exposure to PCBs has been comprehensively monitored through extensive biomonitoring programmes in Sweden, U.S.A, Germany, and Japan, and research has concluded that PCBs not only accumulate effectively in humans, but also contribute to a long-term toxic exposure that increases with age (Link et al., 2005; Minh et al., 2006; Noren and Meironyte, 2000; Patterson et al., 2009). Since the ban and regulation of PCBs was made effective during the 1970s, most temporal studies have observed declining trends of PCBs in the general population (Fangstrom et al., 2008; Hardell et al., 2010). However, despite the declining concentrations in the general population, evidence is growing that even the quite low levels seen today might have a negative effect on human health. For example, several studies have linked levels of PCBs with cardiovascular disease, type-2 diabetes, and obesity (Lee et al., 2010; Son et al., 2010; Turyk et al., 2007). Still, there is very little information regarding the mechanisms behind these observations.

Like other lipophilic exogenous compounds, PCBs have to be metabolised in order to be excreted. A first oxidative phase I reaction step is carried out by the members of the cytochrome P450 system. Several CYPs included in that system have been described to interfere with PCBs, such as CYP1A1/2, CYP3A4, CYP2A6, CYP2B6, CYP2C19 (Gährs et al., 2013; Lehmler et al., 2010). Oxidised PCBs can later undergo phase II metabolism to glucuronic or sulphate conjugates, as recently reviewed by Kania-Korwel and Lehmler.

Recently, genotyping, which aims at characterising the genetic variation within and between different human populations, has become increasingly important in the quest to understand the mechanism behind the complex processes underlying disease susceptibility. From this perspective, genome-wide association studies (GWAS) offer new possibilities to study how genetic variation might influence the metabolism of PCBs and thereby identify individuals that are susceptible to their toxicity. So far, only a few studies have examined the associations between different organic pollutants and genetic variation in human populations. These studies have, for example, found PCBs to be influenced by the CYP1A1 gene (Lind et al., 2014), while a polybrominated diphenylether (PBDE47) was found to be associated with the CYP2B6 (Penell et al., 2014). Another study of 290 individuals exposed to perfluorooctane sulphonic acid (PFOS) and perfluorooctanoic acid (PFOA) found several genetic variants involved in cholesterol metabolism to be associated to these perfluorinated compounds in an inverse manner (Fletcher et al., 2013).

The aim of this study is to examine the associations between plasma levels of a broad range of PCBs, including seven dioxin-like PCBs, and the genetic variability among 1016 individuals using a genome-wide association approach. For environmental contaminants with a long half-life, like the PCBs, a plasma sample is good estimate of exposure.

## 2 Materials and methods

### 2.1 Study population

The Prospective Investigation of Vasculature in Uppsala Seniors (PIVUS) study was initiated in 2001 to investigate the predictive power of different measurements of vascular characteristics for future cardiovascular events. Secondary aims of the study included measurements of cardiac and metabolic function, as well as serum biomarkers and levels of environmental pollutants, including PCBs. All individuals aged 70 and living in the community of Uppsala in Sweden were deemed eligible for the study. The subjects were selected from the community register and invited to participate in a randomised order between April 2001 and June 2004. They received an invitation letter for participation within 2 months of their 70th birthday. Of the 2025 subjects invited, 1016 (507 male, 509 female) subjects agreed to participate. The participants were asked to complete a questionnaire about their medical history, smoking habits and regular medication. Sample details and methods are described more fully elsewhere (Lind et al., 2005).

### 2.2 Chemical analysis

Polychlorinated biphenyls 2,2′,4,4′,5-pentachlorobiphenyl (PCB-74), 2,2′,4,4′,5-pentachlorobiphenyl (PCB-99), 2,3,3′,4,4′-pentachlorobiphenyl (PCB-105), 2,3′,4,4′,5-pentachlorobiphenyl (PCB-118), 3,3′,4,4′,5-pentachlorobiphenyl (PCB126), 2,2′,3,4,4′,5′-Hexachlorobiphenyl (PCB-138), 2,2′,4,4′,5,5′-Hexachlorobiphenyl (PCB-153), 2,3,3′,4,4′,5-Hexachlorobiphenyl (PCB-156), 2,3,3′,4,4′,5′-Hexachlorobiphenyl (PCB-157), 3,3′,4,4′,5,5′-Hexachlorobiphenyl (PCB-169), 2,2′,3,4,4′,5,5′-Heptachlorobiphenyl (PCB-70), 3,3′,4,4′,5,5′-Hexachlorobiphenyl (PCB-180), 2,3,3′,4,4′,5,5′-Heptachlorobiphenyl (PCB-189), 2,2′,3,3′,4,4′,5,5′-Octachlorobiphenyl (PCB-194), 2,2′,3,3′,4,4′,5,5′,6-Nonachlorobiphenyl (PCB-206), and (2,2′,3,3′,4,4′,5,5′,6,6′-Decachlorobiphenyl (PCB-209) were measured in 1016 stored plasma samples. Briefly, frozen plasma was allowed to thaw at room temperature and formic acid was added to 500 µL plasma samples to denature proteins, after which sonication was performed. Internal standards (^13^C-labelled 2,3′,4′,5-Tetrachlorobiphenyl (^13^C-PCB-70), 2,2′,4,5,5′-pentachlorobiphenyl (^13^C-PCB-101), ^13^C-PCB-118, ^13^C-PCB-105, ^13^C-PCB-138, ^13^C-PCB-153, ^13^C-PCB-156, ^13^C-PCB-170, ^13^C-PCB-180, ^13^C-PCB-194, ^13^C-PCB-206, ^13^C-PCB-209) were added to the samples and sonicated prior to the solid-phase extraction (SPE) using Oasis® HLB single use cartridges (6 cm^3^/150 mg, Waters, Milford, MA, USA). The extracts were further purified with multi-layered acidified silica columns containing 56% KOH silica gel, 40% H_2_SO_4_ silica gel and activated anhydrous Na_2_SO_4_. Of the final 25 µL volume (in toluene), 2 µL was injected on a HRGC/HRMS system and measurements were performed on a Micromass Autospec Ultima (Waters, Mildford, MA, USA) mass spectrometer (MS) operating at ≥10,000 resolving power using EI ionisation at 35 eV. Measurements were performed in selective ion mode (SIM) and quantification was performed according to the isotope dilution method using the ^13^C-labelled standards. The MS was coupled to a 6890N gas chromatograph (GC) (Agilent Technologies, Atlanta, GA, USA). The injector was programmed to 275 °C. The PCBs were separated on a 30 m×0.25 i.d.×0.25 µm DB-5 capillary column (SGE Analytical Science, Victoria, AUS) with the GC oven programmed from 180 °C (2 min) to 260 °C (3.5 °C/min) and to 300 °C (6.5 °C/min, 2 min). The analytical method applied to all plasma samples was successfully validated in terms of recovery, precision, and reproducibility. Recovery of labelled internal standards ranged between 49–86%. Reproducibility of procedural quality control samples was below 25% relative standard deviation for PCBs above the limit of detection in the samples. Contamination of procedural blank samples did not exceed any PCB levels above 5% of the levels in the authentic samples. Limits of detection were between 0.8–117.7 pg/mL. Further details on the method and its analytical performance have been described by previously (Salihovic et al., 2012b).

### 2.3 Genotype analysis

DNA samples were genotyped according to the manufacturer's instructions on Illumina Infinium Omni Express bead microarrays. Samples were excluded from downstream analyses if the call rate was less than 95%, if they had extreme heterozygosity (>3 SD from the mean), if they were ethnic outliers, or if they were gender discordant. SNP quality control measures included exact *p*-value for deviation from Hardy–Weinberg equilibrium (HWE) <10^−6^ and missing genotype rate>0.01 (minor allele frequency <5%) or missing genotype rate >0.05 (minor allele frequency≥5%). Multidimensional scaling was performed to obtain principal components to adjust for population structure. Prior to imputation, variants with minor allele frequency (MAF) <1% were removed from the GWAS scaffold. Samples were pre-phased with SHAPEIT2. Genotype data were then imputed up to the “all ancestries” reference panel from the 1000 Genomes Project Consortium Phase 1 interim release (June 2010) (Abecasis et al., 2012) using IMPUTEv2 (Howie et al., 2012).

### 2.4 Statistical analysis

Wet-weight plasma concentrations (pg/mL) of polychlorinated biphenyls were inverse rank normalised to generate a Gaussian distribution for downstream association analyses, and minimise the impact of outliers. Association testing for each transformed PCB congener was performed in a linear regression framework under an additive model in the minor allele, after adjusting for triglycerides, cholesterol, gender and 2 principle components from multidimensional scaling to account for population structure as covariates. This is performed because lipids in the circulation transport PCBs. In addition, there are gender differences between PCBs (Salihovic et al., 2012a). Age was not included as a covariate because all individuals were of the same age.

Association testing was performed in SNPTEST, allowing for uncertainty in the imputation in a missing data likelihood. The association analysis was restricted to SNPs with MAF >1%, imputation quality information score (info) of greater than 0.4, and HWE exact *p*<10^−10^. The genomic control inflation factor for each PCB congener was used to assess evidence of residual population structure (Devlin and Roeder, 1999). It is calculated by converting p values to chi square and taking the median value of the SNPs analysed. To account for multiple testing of 16 PCBs, we defined genome-wide significance as *p*<3.1×10^−9^. Conditional analyses to search for secondary signals of association were performed by including genotype dosage at the lead SNP as an additional covariate in the linear regression model. To evaluate the impact of genetic variation on multiple pollutants, we also performed reciprocal conditional phenotype analysis, by including serum levels of one PCB as an additional covariate in the regression model for another. SNPs that passed genome wide significance were annotated with the Encyclopaedia for DNA elements (ENCODE, 2011) using Annovar (Wang et al., 2010).

## 3 Results

In total, PCBs were successfully determined in 922 participants and the majority of the studied congeners were detected in 70–100% of the samples. Median values for the studied PCBs are presented in Table 1. Among the 16 PCB congeners measured, PCB-153 was detected in the highest plasma concentrations among the participants (1430 pg/ml) followed by PCB-180, -138, and -118. Together, these four compounds accounted for >70% of the entire PCB exposure in the participants. Compared to other studies, the concentrations of the PCBs in the present study were found similar, or comparable, to other studies of general populations from Sweden and Europe (Salihovic et al., 2012a).

**Fig. 1:**
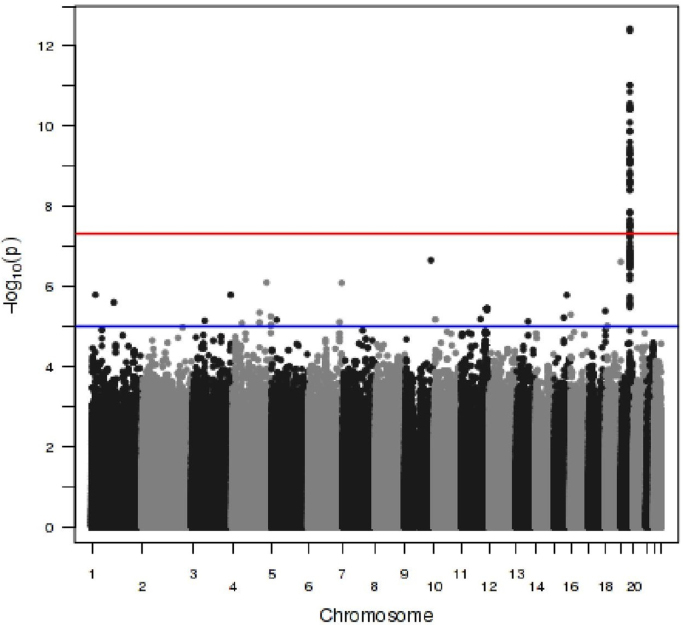
Manhattan plot of PCB 99. Each point corresponds to a SNP passing QC, plotted according to genomic position on the *x*-axis and the strength of association (−log10 *p*-value) on the *y*-axis. The red line indicates the genome wide significance threshold (5×10^−8^), while the blue line indicates a threshold of 10^−05^. (For interpretation of the references to colour in this figure legend, the reader is referred to the web version of this article.)

**Fig. 2:**
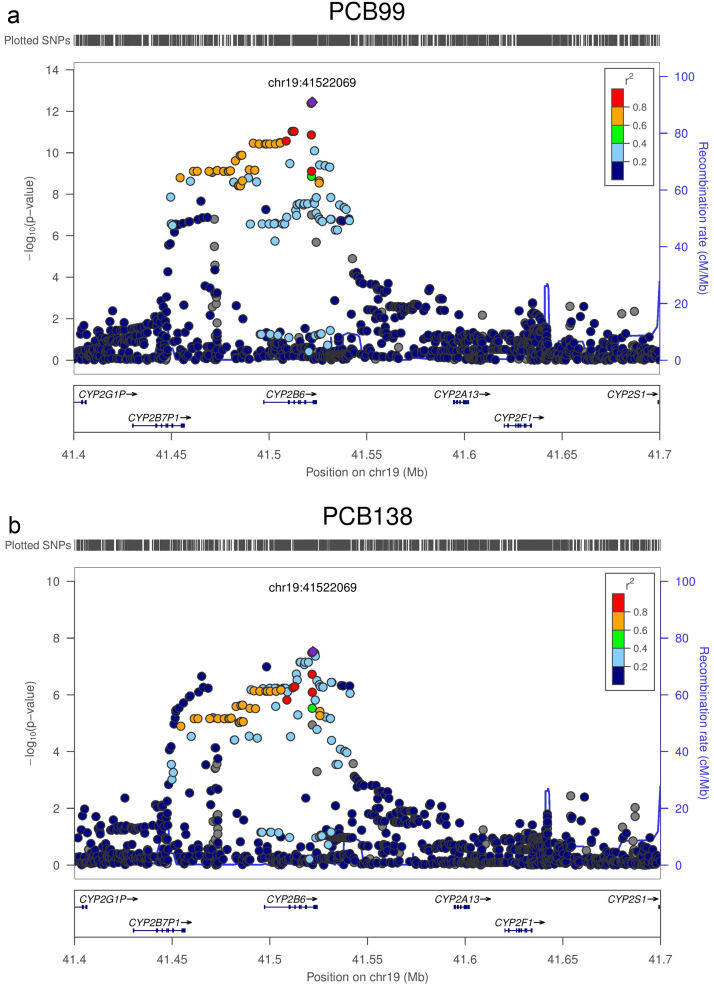
(a) Regional Association plot of PCB 99. Each point represents a SNP plotted with their *p*-value (on a −log10 scale) as a function of genomic position (NCBI Build 37). In each panel, the lead SNP is represented by the purple diamond. The colour coding of all other SNPs (circles) indicates LD with the lead SNP (estimated by CEU *r*^2^ from the 1000 Genomes Project March 2012 release): red *r*^2^≥0.8; gold 0.6≤*r*^2^<0.8; green 0.4≤*r*^2^<0.6; cyan 0.2≤*r*^2^<0.4; blue *r*^2^<0.2; grey *r*^2^ unknown. Recombination rates are estimated from the International HapMap Project and gene annotations are taken from the University of California Santa Cruz genome browser. (b) Regional Association plot of PCB 138. Each point represents a SNP plotted with their *p*-value (on a −log10 scale) as a function of genomic position (NCBI Build 37). In each panel, the lead SNP is represented by the purple diamond. The colour coding of all other SNPs (circles) indicates LD with the lead SNP (estimated by CEU *r*^2^ from the 1000 Genomes Project March 2012 release): red *r*^2^≥0.8; gold 0.6≤*r*^2^<0.8; green 0.4≤*r*^2^<0.6; cyan 0.^2^≤*r*^2^<0.4; blue *r*^2^<0.2; grey *r*^2^ unknown. Recombination rates are estimated from the International HapMap Project and gene annotations are taken from the University of California Santa Cruz genome browser. (For interpretation of the references to colour in this figure legend, the reader is referred to the web version of this article.)

Following imputation, a total of 8,736,858 high-quality SNPs with MAF >1% were included in the analysis. The genomic control inflation factor for each PCB congener was close to 1 (Table 1), so no additional correction for residual population structure after adjustment for principal components was required. We identified genome-wide significant evidence of association for plasma levels of PCB-99, mapping to *CYP2B6*. The SNP with the strongest association signal was rs8109848 (*β*=0.34, SE=0.05, *p*=3.7×10^−13^), which maps to an intronic region of *CYP2B6* (Fig. 1 and 2, Supplemental Tables S1 and S2). This SNP also showed weaker association with several other PCBs (Supplemental Table S3) including PCB-138 (*p*=3.0×10^−8^) and PCB-153 (*p*=0.000041). Conditional analyses, including the genotype dosage of rs8109848 as a covariate in the regression model, extinguished the association signal for all PCBs at this locus.

**Fig. 3:**
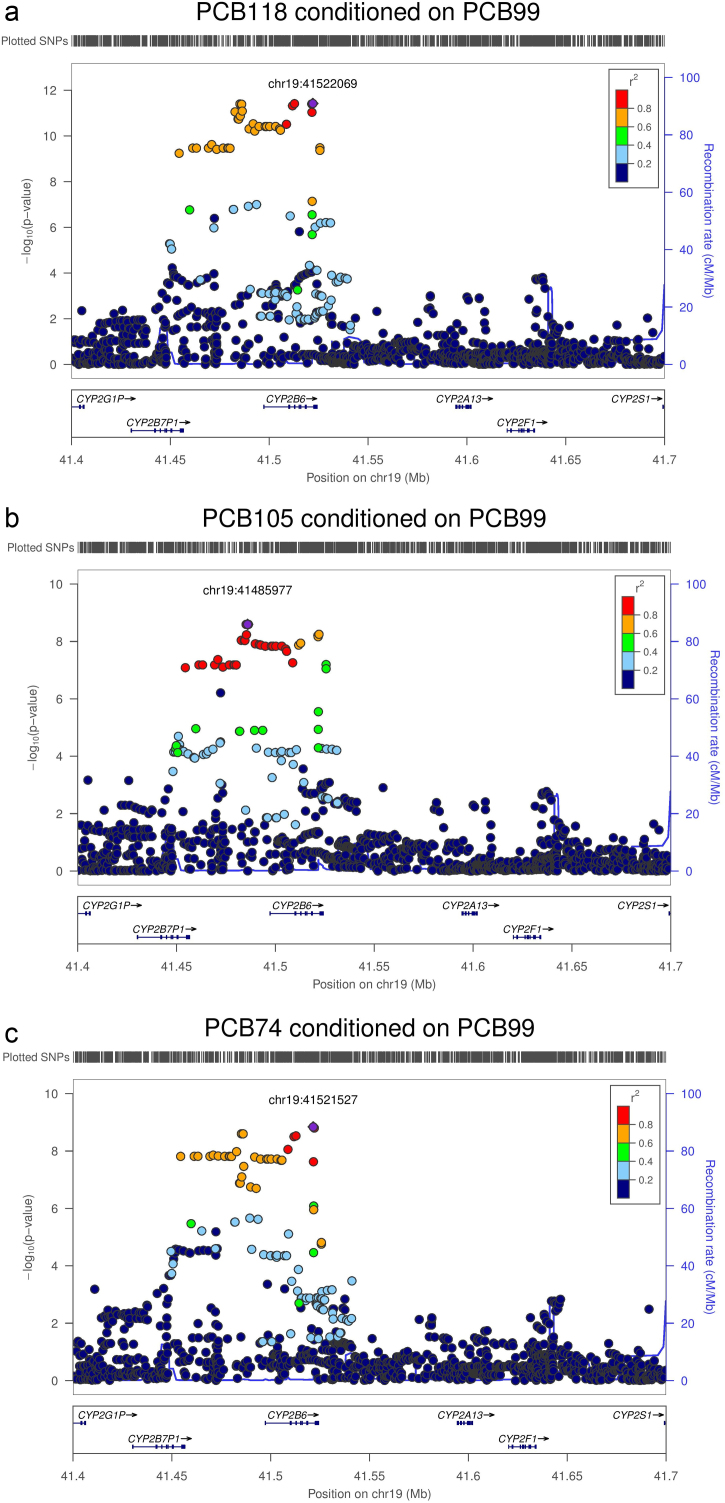
(a) Regional Association plot of PCB118 conditioned on PCB 99. Each point represents a SNP plotted with their *p*-value (on a −log10 scale) as a function of genomic position (NCBI Build 37). In each panel, the lead SNP is represented by the purple diamond. The colour coding of all other SNPs (circles) indicates LD with the lead SNP (estimated by CEU *r*^2^ from the 1000 Genomes Project March 2012 release): red *r*^2^≥0.8; gold 0.6≤*r*^2^<0.8; green 0.4≤*r*^2^<0.6; cyan 0.2≤*r*^2^<0.4; blue *r*^2^<0.2; grey *r*^2^ unknown. Recombination rates are estimated from the International HapMap Project and gene annotations are taken from the University of California Santa Cruz genome browser. (b) Regional Association plot of PCB 105 conditioned on PCB 99. Each point represents a SNP plotted with their *p*-value (on a −log10 scale) as a function of genomic position (NCBI Build 37). In each panel, the lead SNP is represented by the purple diamond. The colour coding of all other SNPs (circles) indicates LD with the lead SNP (estimated by CEU *r*^2^ from the 1000 Genomes Project March 2012 release): red *r*^2^≥0.8; gold 0.6≤*r*^2^<0.8; green 0.4≤*r*^2^<0.6; cyan 0.2≤*r*^2^<0.4; blue *r*^2^<0.2; grey *r*^2^ unknown. Recombination rates are estimated from the International HapMap Project and gene annotations are taken from the University of California Santa Cruz genome browser. (c) Regional Association plot of PCB 74 conditioned on PCB 99. Each point represents a SNP plotted with their *p*-value (on a −-log10 scale) as a function of genomic position (NCBI Build 37). In each panel, the lead SNP is represented by the purple diamond. The colour coding of all other SNPs (circles) indicates LD with the lead SNP (estimated by CEU *r*^2^ from the 1000 Genomes Project March 2012 release): red *r*^2^≥0.8; gold 0.6≤*r*^2^<0.8; green 0.4≤*r*^2^<0.6; cyan 0.2≤*r*^2^<0.4; blue *r*^2^<0.2; grey *r*^2^ unknown. Recombination rates are estimated from the International HapMap Project and gene annotations are taken from the University of California Santa Cruz genome browser. (For interpretation of the references to colour in this figure legend, the reader is referred to the web version of this article.)

Given the known high correlation between different pollutant levels (Lampa et al., 2012) (Supplemental Fig. 1), we sought to dissect the effect of *CYP2B6* variation on PCB-99 and other PCB congeners through reciprocal conditional phenotype analyses. After adjusting for PCB-99 as an additional covariate in the regression model, the previously observed associations of rs8109848 with PCB-138 and PCB-153 were extinguished (*p*=0.17 and *p*=0.0074 respectively). These data would suggest that the association of rs8109848 with PCB-138 and PCB-153 is mediated through PCB-99, and does not reflect pleiotropic effects on serum levels of the pollutants. We next performed conditional phenotype association testing for all other PCB congeners within the *CYP2B6* region, adjusting for PCB-99 levels as an additional covariate in the regression model. When all PCBs were conditioned on PCB-99, further genome-wide significant association signals were revealed for PCB-74, PCB-105 and PCB-118, all mapping to the same genomic region (Fig. 3). The lead SNPs were rs8109848 (*β*=−0.21, SE=0.03, *p*=3.8×10^−12^) for PCB118, rs4802104 (*β*=−0.19, SE=0.03, *p*=1.4×10^−9^) for PCB-74 and rs4803413 (*β*=−0.18, SE=0.03, *p*=2.5×10^−9^) for PCB 105. These SNPs had no significant association in unconditional analysis: rs8109848 (*p*=0.43) for PCB-118, rs4802104 (*p*=0.32) for PCB-74 and rs4803413 (*p*=0.27) for PCB-105.

The three index SNPs are in strong linkage disequilibrium (LD) in European ancestry populations (*r*^2^>0.76), and thus may reflect the same underlying causal variant. Furthermore, when the PCBs were conditioned on PCB-99 and PCB-118 together, the association signals at this locus were extinguished for PCB-74 (rs4802104, *p*=0.02) and PCB-105 (rs4803413, *p*=0.52). Furthermore, no additional signals of association achieved genome-wide significance.

Taken together, these findings suggest that the identified association signals observed for PCBs at this locus are mediated through PCB-99 and PCB-118. Annotation with ENCODE database revealed that several SNPs associated with serum levels of PCBs at the *CYP2B6* locus are located in H3k27me3 and H3k4me1 regions. H3K27Me3 is known to repress transcription, while H3K4Me1 is known to mark active enhancers, suggesting two potential functional mechanisms for serum PCB metabolism at this locus.

## 4 Discussion

This study demonstrates, through a GWAS approach, an association between genetic variation mapping to the *CYP2B6* locus and plasma levels of multiple PCBs within a population-based cohort. The relationship appears to be complex, with effects mediated predominantly through PCB-99 and PCB-118.

The present study is the first examination, in humans, of the relationship between genome-wide genetic variation and plasma levels of a broad range of PCBs. Our results support the view that *CYP2B6* is involved in the metabolism of at least some PCBs in humans, as previously described in experimental studies (Gährs et al., 2013; Lehmler et al., 2010). Furthermore, CYP2B6 has also been reported to metabolise a range of environmental chemicals, including insecticides such as malathion (Buratti et al., 2005) and carbofuran (Usmani et al., 2004).

*CYP2B6* has several known alleles (http://www.cypalleles.ki.se/CYP2B6.htm), many of which are associated with serum levels of a range of pharmaceutical drugs. *CYP2B6* genotyping has been applied successfully in HIV-infected individuals to reduce the therapeutic dose of efavirenz, resulting in improvement of central nervous system-related side effects (Gatanaga et al., 2007). The commonest allele is *CYP2B6*⁎6 [Q172H and K262R], tagged by rs3745274 and rs2279343, respectively. However, these tagging SNPs were not associated with serum PCB levels in the present study.

All PCBs found to be associated with genetic variation in CYP2B6 in this study are highly bioaccumulative. One common way to characterize the PCBs is through their binding to the aryl hydrocarbon (Ah)-receptor. Because of their structure, dioxin-like PCBs are agonists of Ah-receptors, while non-dioxin-like PCBs are not. PCB-99, PCB-138 and PCB-153 are non-dioxin like, while PCB-118 and PCB-105 are dioxin-like. McFarland and Clarke (1989) suggested a classification based on the ability to induce CYPs in a barbiturate-like fashion, and PCB-99 and PCB-153 belong to that class. Wolff et al. later further subdivided the PCBs in to antiestrogenenic and phenobarbital-type inducers, and PCB-99 and PCB-153 belong to that latter class (Wolff et al., 1997). It should, however, be pointed out that not all PCBs considered to be phenobarbital-type inducers demonstrated an association with genetic variation in the *CYP2B6* gene in the present study. Within this group it was mainly those with a moderate number of chlorine atoms that were associated, but not those with a large number of chlorine atoms, such as PCB-180 and PCB-194, having a longer half-life (Wimmerova et al., 2011).

Conditional phenotype analyses suggest that association signals with pollutants at the *CYP2B6* locus are mediated through PCB-99 and PCB-118. The only common denominator for these pollutants, being related to variation in the *CYP2B6* gene, seems to be the low number of chlorine substitutions, possibly reflecting a shorter half-life than the more lipophilic PCBs with a larger number of chlorine atoms. The substitution pattern of PCB-99 and PCB-118 with chlorine substitution in both the meta- and the para-position prevents degradation in biota (Furukawa et al., 1978). The same is true for the hexachlorobiphenyls PCB-153 and -138. The larger molecules, hepta- to decacholorbiphenyl congeners and dioxin-like hexachlorobiphenyl congeners show no correlation vs variation in the *CYP2B6* gene.

## 5 Conclusions

The present study is the first of its kind to relate genetic variation to plasma concentrations of PCBs by means of a GWAS approach. This approach has the advantage that it covers the information on variation in all human genes, and thereby no prior hypotheses on which genes that are of importance are needed. The environmental implication of the current results is that the CYP2B6 is involved in PCB metabolism in humans and that subjects with a certain CYP2B6 genotype might be more susceptible for the toxic effects of PCBs.

## Figures and Tables

**Table 1:** Median concentrations (pg/mL), with 25th and 75th percentiles and genomic control lambda are given for the different PCBs included in the study.

Pollutant	Median value	Genomic control lambda
PCB-74	91.4	1.000
2,2′,4,4′,5-pentachlorobiphenyl	(63.9–128.1)	
PCB-99	90.9	0.995
2,2′,4,4′,5-pentachlorobiphenyl	(62.4–131.9)	
PCB-105	32.0	1.001
2,3,3′,4,4′-pentachlorobiphenyl	(21.0–46.8)	
PCB-118	200.6	0.998
2,3′,4,4′,5-pentachlorobiphenyl	(136.4–281)	
PCB-126	40.4	1.005
3,3′,4,4′,5-pentachlorobiphenyl	(71.9–385.8)	
PCB-138	819.3	1.001
2,2′,3,4,4′,5′-Hexachlorobiphenyl	(619.2–1115.8)	
PCB-153	1427.6	0.996
2,2′,4,4′,5,5′-Hexachlorobiphenyl	(1114.4–1847.9)	
PCB-156	154.3	1.007
2,3,3′,4,4′,5-Hexachlorobiphenyl	(118.7–197.6)	
PCB-157	28.0	1.015
2,3,3′,4,4′,5′-Hexachlorobiphenyl	(21.4–37.0)	
PCB169	171.4	0.997
3,3′,4,4′,5,5′-Hexachlorobiphenyl	(219.9–636.4)	
PCB-170	497.5	1.002
2,2′,3,4,4′,5,5′-Heptachlorobiphenyl	(385.7–633.0)	
PCB-180	1165.4	1.001
3,3′,4,4′,5,5′-Hexachlorobiphenyl	(917.8–1487.8)	
PCB-189	19.3	0.999
2,3,3′,4,4′,5,5′-Heptachlorobiphenyl	(14.6–25.8)	
PCB194	119.4	1.003
2,2′,3,3′,4,4′,5,5′-Octachlorobiphenyl	(87.6–158.9)	
PCB-206	26.8	0.987
2,2′,3,3′,4,4′,5,5′,6-Nonachlorobiphenyl	(20.8–35.2)	
PCB-209	26.2	0.982
(2,2′,3,3′,4,4′,5,5′,6,6′-Decachlorobiphenyl)	(19.6–34.7)	

## Footnotes

Appendix A Supplementary data associated with this article can be found in the online version at doi:10.1016/j.envres.2015.03.022.
