# Supplementary material for: Genome-wide association study of plasma levels of polychlorinated biphenyls disclose an association with the CYP2B6 gene in a population-based sample
Source: Environ Res. 2015 Jul;140:95–101. doi: 10.1016/j.envres.2015.03.022 (PMC4509719; doi:10.1016/j.envres.2015.03.022)

Supplemental Material

Genome Wide Association Study of Polychlorinated Biphenyls (PCBs)

Esther Ng^1^, P. Monica Lind^2^, Anubha Mahajan^1^, Anne-Christine Syvänen^2^, Tomas Axelsson^2^, Erik Ingelsson^1,3^, Cecilia M. Lindgren^1^, Samira Salihovic^5^, Bert van Bavel^5^, Andrew P. Morris^1,4^, Lars Lind^2^

1. Wellcome Trust Centre for Human Genetics, University of Oxford, Oxford, United Kingdom
2. Department of Medical Sciences, Uppsala University, 751 85 Uppsala, Sweden
3. Department of Medical Sciences, Molecular Epidemiology and Science for Life Laboratory, Uppsala University, Uppsala, Sweden
4. Department of Biostatistics, University of Liverpool, Liverpool, United Kingdom
5. MTM Research Center, Örebro University, 701 82 Örebro, Sweden

Table of Contents

1. Supplemental Table S1 Lead SNPs for each pollutant with p value below 10^-5^
2. Supplemental Table S2 SNPs which pass the genome wide significance threshold of 3.1x10^-8^
3. Supplemental Table S3 p value of rs8109848 for each pollutant
4. Supplemental Table S4 Encode histone mark annotations for SNPs which pass the genome wide significance threshold of 3.1x10^-8^
5. Supplemental Figure 1 Hierarchical clustering of pollutant levels based on Euclidean distance metric

Supplemental Table S1 Index SNPs with nominal evidence of association (*p*<10^-6^) for each PCB

| PCB | Chr | Coordinate | MAF | p-value | beta | SE | rsid |
| --- | --- | --- | --- | --- | --- | --- | --- |
| pcb105 | 6 | 164452105 | 0.023 | 4.2E-08 | -1.03 | 0.19 | rs62437588 |
| pcb105 | 8 | 7195467 | 0.019 | 1.5E-07 | -1.32 | 0.25 | rs188324495 |
| pcb105 | 15 | 83072199 | 0.037 | 2.4E-07 | -0.73 | 0.14 |  |
| pcb118 | 6 | 164452105 | 0.023 | 1.1E-07 | -1.02 | 0.19 | rs62437588 |
| pcb118 | 8 | 6933373 | 0.046 | 5.6E-07 | -0.81 | 0.16 | rs117880470 |
| pcb118 | 15 | 83072199 | 0.037 | 6.6E-07 | -0.71 | 0.14 |  |
| pcb118 | 15 | 78175364 | 0.074 | 7.1E-07 | 0.56 | 0.11 |  |
| pcb118 | 18 | 70129472 | 0.021 | 8.3E-07 | 0.80 | 0.16 | rs117120368 |
| pcb126 | 1 | 92169004 | 0.25 | 1.9E-07 | 0.28 | 0.05 | rs3767574 |
| pcb126 | 2 | 195199595 | 0.024 | 2.9E-08 | -1.23 | 0.22 | rs62196198 |
| pcb126 | 14 | 32225501 | 0.47 | 7.7E-07 | -0.23 | 0.05 | rs77828254 |
| pcb138 | 3 | 18721736 | 0.019 | 1.5E-07 | -1.11 | 0.21 | rs76942353 |
| pcb138 | 4 | 167731888 | 0.023 | 2.8E-07 | -0.95 | 0.18 | rs143200770 |
| pcb138 | 6 | 164452105 | 0.023 | 1.9E-07 | -0.96 | 0.18 | rs62437588 |
| pcb138 | 19 | 41522069 | 0.31 | 3.0E-08 | 0.26 | 0.05 | rs8109848 |
| pcb153 | 3 | 104281080 | 0.021 | 4.6E-07 | -0.76 | 0.15 | rs140092704 |
| pcb153 | 3 | 18721736 | 0.019 | 5.6E-07 | -1.02 | 0.20 | rs76942353 |
| pcb153 | 6 | 164452105 | 0.023 | 7.2E-07 | -0.94 | 0.19 | rs62437588 |
| pcb153 | 19 | 41523016 | 0.40 | 1.1E-07 | 0.23 | 0.04 | rs3181842 |
| pcb156 | 3 | 159268463 | 0.024 | 9.1E-07 | 1.04 | 0.21 | rs7616179 |
| pcb156 | 11 | 105277570 | 0.013 | 2.3E-07 | 1.29 | 0.25 | rs117325735 |
| pcb156 | 11 | 34881635 | 0.022 | 5.3E-07 | -1.15 | 0.23 | rs146771634 |
| pcb156 | 16 | 20543740 | 0.48 | 2.2E-07 | -0.26 | 0.05 |  |
| pcb157 | 16 | 20543740 | 0.48 | 4.9E-08 | -0.28 | 0.05 |  |
| pcb157 | 21 | 43482046 | 0.48 | 5.9E-07 | -0.22 | 0.04 | rs220262 |
| pcb169 | 1 | 156827703 | 0.015 | 2.9E-07 | -1.21 | 0.24 | rs115699453 |
| pcb169 | 2 | 80794915 | 0.012 | 3.0E-08 | -1.73 | 0.31 | rs191655746 |
| pcb169 | 4 | 83093230 | 0.083 | 1.3E-07 | -0.51 | 0.10 | rs182442984 |
| pcb169 | 14 | 51326166 | 0.293 | 4.3E-07 | 0.25 | 0.05 | rs1953884 |
| pcb170 | 1 | 9673843 | 0.011 | 3.2E-07 | -1.55 | 0.30 | rs11121451 |
| pcb170 | 2 | 36865755 | 0.010 | 2.6E-07 | -1.62 | 0.32 | rs74853015 |
| pcb170 | 4 | 83093230 | 0.083 | 1.9E-07 | -0.48 | 0.09 | rs182442984 |
| pcb170 | 4 | 169706997 | 0.23 | 8.4E-07 | -0.26 | 0.05 | rs113026070 |
| pcb170 | 9 | 6643981 | 0.016 | 5.2E-07 | -1.27 | 0.25 | rs150484890 |
| pcb170 | 13 | 57119253 | 0.010 | 4.9E-07 | -1.64 | 0.33 | rs117093004 |
| pcb170 | 13 | 61532597 | 0.23 | 9.5E-07 | 0.25 | 0.05 | rs1847505 |
| pcb180 | 1 | 9673843 | 0.011 | 1.4E-07 | -1.66 | 0.31 | rs11121451 |
| pcb180 | 4 | 83093230 | 0.083 | 4.1E-07 | -0.48 | 0.09 | rs182442984 |
| pcb180 | 13 | 80845641 | 0.033 | 1.4E-07 | -0.62 | 0.12 | rs171156 |
| pcb189 | 4 | 71183979 | 0.013 | 1.1E-07 | 1.46 | 0.27 | rs17714883 |
| pcb189 | 7 | 85048380 | 0.017 | 5.2E-07 | 0.94 | 0.19 | rs142857449 |
| pcb189 | 9 | 5972759 | 0.019 | 2.7E-07 | 1.23 | 0.24 | rs139863644 |
| pcb194 | 2 | 33776680 | 0.23 | 7.4E-07 | 0.26 | 0.05 | rs2367809 |
| pcb194 | 2 | 149086644 | 0.010 | 9.8E-07 | -1.19 | 0.24 | rs187650835 |
| pcb194 | 3 | 148756825 | 0.022 | 1.8E-07 | 0.81 | 0.15 | rs6764352 |
| pcb194 | 4 | 118035559 | 0.013 | 9.9E-08 | -1.27 | 0.24 | rs114452217 |
| pcb194 | 8 | 71579910 | 0.032 | 5.2E-07 | -0.74 | 0.15 | rs72667712 |
| pcb194 | 12 | 115468959 | 0.011 | 3.9E-07 | -1.3 | 0.26 | rs144700679 |
| pcb194 | 18 | 4395500 | 0.011 | 6.6E-07 | -1.53 | 0.31 | rs146408330 |
| pcb194 | 18 | 22958783 | 0.014 | 8.6E-07 | -1.03 | 0.21 | rs185818984 |
| pcb206 | 1 | 9674172 | 0.012 | 7.3E-08 | -1.74 | 0.32 | rs11121452 |
| pcb206 | 3 | 134054697 | 0.010 | 8.0E-07 | -1.26 | 0.26 | rs186248261 |
| pcb206 | 7 | 76249289 | 0.019 | 9.2E-07 | 1.22 | 0.25 |  |
| pcb206 | 14 | 21207775 | 0.026 | 6.7E-07 | -0.76 | 0.15 | rs80073712 |
| pcb206 | 16 | 20543740 | 0.483 | 1.3E-07 | -0.27 | 0.05 |  |
| pcb209 | 2 | 3677227 | 0.010 | 2.7E-07 | -1.53 | 0.30 | rs147056028 |
| pcb74 | 1 | 237985697 | 0.011 | 8.3E-07 | -1.54 | 0.31 |  |
| pcb74 | 3 | 18721736 | 0.019 | 5.0E-08 | -1.18 | 0.22 | rs76942353 |
| pcb74 | 3 | 123137562 | 0.014 | 4.0E-07 | 1.29 | 0.25 | rs151013628 |
| pcb74 | 5 | 46117611 | 0.021 | 1.2E-07 | -1.17 | 0.22 | rs77655443 |
| pcb74 | 9 | 126555988 | 0.011 | 6.5E-07 | -1.36 | 0.27 | rs183433444 |
| pcb74 | 15 | 83072199 | 0.037 | 2.7E-07 | -0.72 | 0.14 |  |
| pcb99 | 4 | 167731888 | 0.023 | 7.9E-07 | -0.96 | 0.19 | rs143200770 |
| pcb99 | 6 | 164452105 | 0.023 | 8.1E-07 | -0.92 | 0.19 | rs62437588 |
| pcb99 | 18 | 75110360 | 0.43 | 2.4E-07 | -0.29 | 0.06 | rs10469074 |
| pcb99 | 19 | 41522069 | 0.31 | 3.7E-13 | 0.34 | 0.05 | rs8109848 |

Supplemental Table S2 SNPs mapping to *CYP2B6* locus (chromosome 19q13.2) which pass genome wide significance threshold

| PCB | Position | Allele1 | Allele2 | MAF | pvalue | Beta | SE | rsID |
| --- | --- | --- | --- | --- | --- | --- | --- | --- |
| pcb99 | 41449603 | T | C | 0.24 | 1.4E-08 | 0.29 | 0.05 | rs4124633 |
| pcb99 | 41454470 | G | A | 0.38 | 1.6E-09 | 0.27 | 0.05 | rs4802099 |
| pcb99 | 41459751 | T | C | 0.23 | 2.4E-09 | 0.31 | 0.05 | rs11667592 |
| pcb99 | 41461318 | G | A | 0.38 | 7.8E-10 | 0.28 | 0.05 | rs11673582 |
| pcb99 | 41463215 | G | C | 0.38 | 7.8E-10 | 0.28 | 0.05 | rs2316319 |
| pcb99 | 41465130 | T | C | 0.36 | 2.2E-08 | 0.25 | 0.04 | rs10417579 |
| pcb99 | 41469279 | G | A | 0.38 | 7.8E-10 | 0.28 | 0.05 | rs12978065 |
| pcb99 | 41470920 | A | G | 0.38 | 8.8E-10 | 0.28 | 0.04 | rs35242531 |
| pcb99 | 41473447 | G | C | 0.38 | 7.2E-10 | 0.28 | 0.05 | rs112639001 |
| pcb99 | 41476707 | C | T | 0.38 | 7.8E-10 | 0.28 | 0.05 | rs59300404 |
| pcb99 | 41476740 | G | A | 0.38 | 7.8E-10 | 0.28 | 0.05 | rs57755489 |
| pcb99 | 41479051 | G | A | 0.38 | 7.8E-10 | 0.28 | 0.05 | rs882808 |
| pcb99 | 41479309 | C | T | 0.38 | 7.8E-10 | 0.28 | 0.05 | rs1104854 |
| pcb99 | 41480217 | G | C | 0.38 | 7.4E-10 | 0.28 | 0.05 | rs11667025 |
| pcb99 | 41481829 | G | A | 0.23 | 2.6E-09 | 0.31 | 0.05 | rs11671151 |
| pcb99 | 41481973 | A | C | 0.23 | 2.6E-09 | 0.31 | 0.05 | rs11671108 |
| pcb99 | 41482742 | C | T | 0.35 | 2.5E-10 | 0.29 | 0.05 | rs7251950 |
| pcb99 | 41484257 | C | G | 0.36 | 3.9E-09 | 0.27 | 0.05 | rs111390768 |
| pcb99 | 41484602 | G | T | 0.36 | 3.8E-09 | 0.27 | 0.05 | rs12971445 |
| pcb99 | 41485194 | C | T | 0.35 | 1.4E-10 | 0.30 | 0.05 | rs1808002 |
| pcb99 | 41485380 | A | T | 0.36 | 3.9E-09 | 0.27 | 0.05 | rs56881024 |
| pcb99 | 41485977 | G | A | 0.35 | 1.3E-10 | 0.30 | 0.05 | rs4803413 |
| pcb99 | 41486082 | T | C | 0.35 | 1.3E-10 | 0.30 | 0.05 | rs4803414 |
| pcb99 | 41486375 | T | C | 0.36 | 2.3E-09 | 0.28 | 0.05 | rs55721612 |
| pcb99 | 41489448 | G | A | 0.23 | 1.6E-09 | 0.31 | 0.05 | rs1808682 |
| pcb99 | 41489851 | T | C | 0.35 | 6.6E-10 | 0.29 | 0.05 | rs892216 |
| pcb99 | 41491918 | A | G | 0.35 | 3.5E-11 | 0.31 | 0.05 | rs8109525 |
| pcb99 | 41492677 | T | C | 0.35 | 7.0E-10 | 0.29 | 0.05 | rs7257854 |
| pcb99 | 41493603 | A | G | 0.23 | 2.5E-09 | 0.31 | 0.05 | rs35659050 |
| pcb99 | 41494891 | T | C | 0.35 | 3.8E-11 | 0.31 | 0.05 | rs7254579 |
| pcb99 | 41497997 | C | T | 0.35 | 3.8E-11 | 0.31 | 0.05 | rs1872122 |
| pcb99 | 41498733 | T | C | 0.35 | 3.8E-11 | 0.31 | 0.05 | rs6508963 |
| pcb99 | 41500213 | T | C | 0.35 | 3.8E-11 | 0.31 | 0.05 | rs8100458 |
| pcb99 | 41503245 | C | T | 0.35 | 3.8E-11 | 0.31 | 0.05 | rs4802103 |
| pcb99 | 41505238 | A | T | 0.35 | 3.3E-11 | 0.31 | 0.05 | rs35177224 |
| pcb99 | 41505653 | G | A | 0.35 | 3.2E-11 | 0.31 | 0.05 | rs2873265 |
| pcb99 | 41505708 | C | T | 0.35 | 3.4E-11 | 0.31 | 0.05 | rs2873264 |
| pcb99 | 41508744 | G | A | 0.32 | 2.7E-11 | 0.31 | 0.05 | rs11672911 |
| pcb99 | 41510636 | G | A | 0.16 | 3.3E-10 | 0.39 | 0.06 | rs33912321 |
| pcb99 | 41511803 | C | G | 0.32 | 9.3E-12 | 0.32 | 0.05 | rs4803418 |
| pcb99 | 41512792 | C | T | 0.32 | 9.3E-12 | 0.32 | 0.05 | rs4803419 |
| pcb99 | 41515483 | G | A | 0.39 | 2.9E-08 | 0.24 | 0.04 | rs2279344 |
| pcb99 | 41515702 | T | C | 0.39 | 2.9E-08 | 0.24 | 0.04 | rs2279345 |
| pcb99 | 41518007 | G | C | 0.39 | 2.9E-08 | 0.24 | 0.04 | rs6508966 |
| pcb99 | 41519715 | A | C | 0.39 | 2.9E-08 | 0.24 | 0.04 | rs11671243 |
| pcb99 | 41521527 | A | G | 0.31 | 4.1E-13 | 0.34 | 0.05 | rs4802104 |
| pcb99 | 41521638 | G | A | 0.32 | 1.4E-11 | 0.32 | 0.05 | rs7260329 |
| pcb99 | 41521704 | C | G | 0.18 | 1.4E-09 | 0.38 | 0.06 | rs3786548 |
| pcb99 | 41521757 | C | G | 0.22 | 7.7E-10 | 0.35 | 0.06 | rs3786552 |
| pcb99 | 41522069 | G | C | 0.31 | 3.7E-13 | 0.34 | 0.05 | rs8109848 |
| pcb99 | 41523016 | C | T | 0.40 | 2.3E-08 | 0.25 | 0.04 | rs3181842 |
| pcb99 | 41523254 | A | G | 0.15 | 8.0E-11 | 0.39 | 0.06 | rs7260525 |
| pcb99 | 41523303 | T | C | 0.39 | 2.8E-08 | 0.25 | 0.04 | rs7246465 |
| pcb99 | 41524153 | T | C | 0.20 | 1.5E-08 | 0.30 | 0.05 | rs1042389 |
| pcb99 | 41525655 | G | A | 0.27 | 2.3E-09 | 0.29 | 0.05 | rs1552220 |
| pcb99 | 41525657 | T | C | 0.27 | 2.9E-09 | 0.29 | 0.05 | rs1552221 |
| pcb99 | 41525904 | T | A | 0.15 | 3.9E-10 | 0.38 | 0.06 | rs1552222 |
| pcb99 | 41528667 | G | A | 0.15 | 4.2E-10 | 0.37 | 0.06 | rs2113103 |
| pcb99 | 41530883 | G | A | 0.15 | 4.9E-10 | 0.37 | 0.06 | rs71358958 |
| pcb99 | 41530980 | G | A | 0.15 | 4.9E-10 | 0.37 | 0.06 | rs34421950 |
| pcb99 | 41531038 | G | A | 0.15 | 4.9E-10 | 0.37 | 0.06 | rs34594610 |
| pcb99 | 41531597 | C | G | 0.19 | 1.4E-08 | 0.31 | 0.05 | rs35930845 |

Supplemental Table S3 Association of rs8109848 for each pollutant

| Pollutant | p_value | Beta | SE |
| --- | --- | --- | --- |
| pcb74 | 0.31 | 0.05 | 0.04 |
| pcb99 | 3.7 E-13 | 0.34 | 0.05 |
| pcb118 | 0.43 | 0.04 | 0.05 |
| pcb105 | 0.067 | 0.09 | 0.05 |
| pcb153 | 0.000041 | 0.19 | 0.05 |
| pcb138 | 3.0 E-08 | 0.26 | 0.05 |
| pcb156 | 0.93 | 0.00 | 0.05 |
| pcb157 | 0.51 | 0.03 | 0.05 |
| pcb180 | 0.12 | 0.07 | 0.04 |
| pcb170 | 0.05 | 0.09 | 0.04 |
| pcb189 | 0.18 | 0.06 | 0.05 |
| pcb194 | 0.12 | 0.07 | 0.05 |
| pcb206 | 0.15 | 0.07 | 0.05 |
| pcb209 | 0.17 | 0.07 | 0.05 |
| pcb126 | 0.063 | 0.09 | 0.05 |
| pcb169 | 0.18 | 0.06 | 0.05 |

Supplemental Table S4 ENCODE histone marks for SNPs which pass the genome wide significance threshold

| Histone_mark | Chr | Pos | A1 | A2 | Pollutant |
| --- | --- | --- | --- | --- | --- |
| H3k27me3 | 19 | 41521527 | A | G | pcb138 |
| H3k27me3 | 19 | 41522069 | G | C | pcb138 |
| H3k27me3 | 19 | 41523016 | C | T | pcb138 |
| H3k27me3 | 19 | 41523303 | T | C | pcb138 |
| H3k27me3 | 19 | 41449603 | T | C | pcb99 |
| H3k27me3 | 19 | 41454470 | G | A | pcb99 |
| H3k27me3 | 19 | 41459751 | T | C | pcb99 |
| H3k27me3 | 19 | 41461318 | G | A | pcb99 |
| H3k27me3 | 19 | 41463215 | G | C | pcb99 |
| H3k27me3 | 19 | 41465130 | T | C | pcb99 |
| H3k27me3 | 19 | 41469279 | G | A | pcb99 |
| H3k27me3 | 19 | 41470920 | A | G | pcb99 |
| H3k27me3 | 19 | 41473447 | G | C | pcb99 |
| H3k27me3 | 19 | 41476707 | C | T | pcb99 |
| H3k27me3 | 19 | 41476740 | G | A | pcb99 |
| H3k27me3 | 19 | 41479051 | G | A | pcb99 |
| H3k27me3 | 19 | 41479309 | C | T | pcb99 |
| H3k27me3 | 19 | 41480217 | G | C | pcb99 |
| H3k27me3 | 19 | 41481829 | G | A | pcb99 |
| H3k27me3 | 19 | 41481973 | A | C | pcb99 |
| H3k27me3 | 19 | 41482742 | C | T | pcb99 |
| H3k27me3 | 19 | 41484257 | C | G | pcb99 |
| H3k27me3 | 19 | 41484602 | G | T | pcb99 |
| H3k27me3 | 19 | 41485194 | C | T | pcb99 |
| H3k27me3 | 19 | 41485380 | A | T | pcb99 |
| H3k27me3 | 19 | 41485977 | G | A | pcb99 |
| H3k27me3 | 19 | 41486082 | T | C | pcb99 |
| H3k27me3 | 19 | 41486375 | T | C | pcb99 |
| H3k27me3 | 19 | 41489448 | G | A | pcb99 |
| H3k27me3 | 19 | 41489851 | T | C | pcb99 |
| H3k27me3 | 19 | 41491918 | A | G | pcb99 |
| H3k27me3 | 19 | 41492677 | T | C | pcb99 |
| H3k27me3 | 19 | 41493603 | A | G | pcb99 |
| H3k27me3 | 19 | 41494891 | T | C | pcb99 |
| H3k27me3 | 19 | 41497997 | C | T | pcb99 |
| H3k27me3 | 19 | 41498733 | T | C | pcb99 |
| H3k27me3 | 19 | 41500213 | T | C | pcb99 |
| H3k27me3 | 19 | 41503245 | C | T | pcb99 |
| H3k27me3 | 19 | 41505238 | A | T | pcb99 |
| H3k27me3 | 19 | 41505653 | G | A | pcb99 |
| H3k27me3 | 19 | 41505708 | C | T | pcb99 |
| H3k27me3 | 19 | 41508744 | G | A | pcb99 |
| H3k27me3 | 19 | 41510636 | G | A | pcb99 |
| H3k27me3 | 19 | 41511803 | C | G | pcb99 |
| H3k27me3 | 19 | 41512792 | C | T | pcb99 |
| H3k27me3 | 19 | 41515483 | G | A | pcb99 |
| H3k27me3 | 19 | 41515702 | T | C | pcb99 |
| H3k27me3 | 19 | 41518007 | G | C | pcb99 |
| H3k27me3 | 19 | 41519715 | A | C | pcb99 |
| H3k27me3 | 19 | 41521527 | A | G | pcb99 |
| H3k27me3 | 19 | 41521638 | G | A | pcb99 |
| H3k27me3 | 19 | 41521704 | C | G | pcb99 |
| H3k27me3 | 19 | 41521757 | C | G | pcb99 |
| H3k27me3 | 19 | 41522069 | G | C | pcb99 |
| H3k27me3 | 19 | 41523016 | C | T | pcb99 |
| H3k27me3 | 19 | 41523254 | A | G | pcb99 |
| H3k27me3 | 19 | 41523303 | T | C | pcb99 |
| H3k27me3 | 19 | 41524153 | T | C | pcb99 |
| H3k27me3 | 19 | 41525655 | G | A | pcb99 |
| H3k27me3 | 19 | 41525657 | T | C | pcb99 |
| H3k27me3 | 19 | 41525904 | T | A | pcb99 |
| H3k27me3 | 19 | 41528667 | G | A | pcb99 |
| H3k27me3 | 19 | 41530883 | G | A | pcb99 |
| H3k27me3 | 19 | 41530980 | G | A | pcb99 |
| H3k27me3 | 19 | 41531038 | G | A | pcb99 |
| H3k27me3 | 19 | 41531597 | C | G | pcb99 |
| H3k4me1 | 19 | 41481829 | G | A | pcb99 |
| H3k4me1 | 19 | 41481973 | A | C | pcb99 |
| H3k4me1 | 19 | 41482742 | C | T | pcb99 |

Supplemental Figure 1


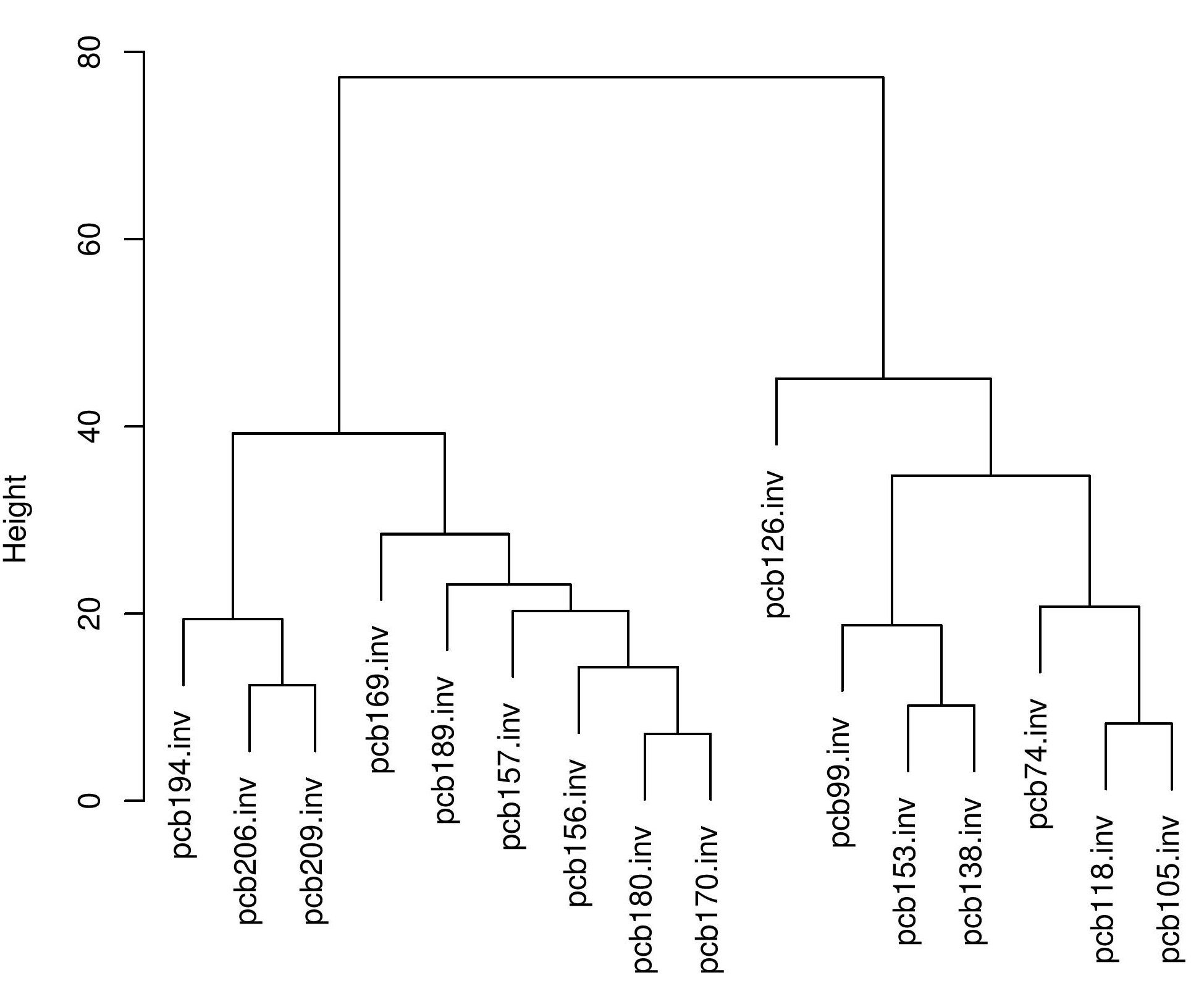

Supplement: Supplementary file 1 — Supplementary material [file mmc1.doc]
